# Validation of the French version of the Vulnerable Elders Survey-13 (VES-13)

**DOI:** 10.1186/s12874-020-0910-x

**Published:** 2020-02-05

**Authors:** Joël Belmin, Lyamna Khellaf, Sylvie Pariel, Witold Jarzebowski, Lucie Valembois, John Zeisel, Carmelo Lafuente-Lafuente

**Affiliations:** 1 Service de gériatrie à orientation cardiovasculaire et neuropsychogériatrique, Hôpital Charles Foix, Assistance Publique-Hôpitaux de Paris, Ivry-sur-Seine, France; 2 Faculté de Médecine, Sorbonne Université, Paris, France; 3 Service de Gériatrie, Hôpital Charles Foix et Université Pierre et Marie Curie, 7 avenue de la République, 94200 Ivry-sur-Seine, France; 4 Service de Gériatrie Ambulatoire, Hôpital Charles Foix, Assistance Publique-Hôpitaux de Paris, Ivry-sur-Seine, France; 5 Service de Gériatrie Centre Hospitalier Général, Bastia, France; 6 Hearthstone Alzheimer Care, Woburn, MA USA

**Keywords:** Frailty, Functional decline, Vulnerability, Mortality, Vulnerable elders survey-13, French version, VES-13, EVA-13

## Abstract

**Background:**

Identifying and assessing degree and type of frailty among older persons is a major challenge when targeting high risk populations to identify preventive interventions. The Vulnerable Elders Survey-(VES-13) is a simple instrument to identify frailty defined as risk for death, functional decline or institutionalization.

**Objective:**

Translate VES-13 into French and validate it.

**Methods:**

The French version of VES-13 was developed by forward-backward translation of the VES-13 survey instrument. The authors assessed its feasibility, construct validity, and ability to predict the combined outcomes of admission to institution or death at 18 months, in 135 persons over 70 years of age living in the community. Subjects were recruited from three settings: Group 1 – a health prevention center (*n* = 45); Group 2 – an ambulatory care geriatric clinic (*n* = 40); and Group 3 – an intermediate care hospital unit (*n* = 50). The combined outcomes data were recorded by telephone interview with participants or a proxy.

**Results:**

Feasibility of the French version, named Echelle de Vulnérabilité des Ainés-13 or EVA-13, was excellent. The scale classified 5 (11%) persons as vulnerable (score of 3 or more) in Group 1, 23 (58%) in Group 2 and 45 (90%) in Group 3 (*p* < 0.001) with scores of 0.91 +/− 1.16, 4.27 +/− 3.17 and 6.90 +/− 3.17, respectively (*p* < 0.001). At follow-up, among the 60 non-vulnerable subjects, 58 (96%) were alive and living at home, whereas 46 (65%) of the 70 vulnerable subjects were alive and living at home (*p* < 0.001).

**Conclusions:**

EVA-13 was determined to be valid and reliable.

## Background

Researchers in the field of gerontology over the past decades, have been increasingly interested in frailty [1–3], a syndrome characterized by age-related vulnerability and decline in functional reserves of physiologic systems. As compared to non-frail older adults, frail elders experience an increased risk of adverse health outcomes such as falls, fractures, hospitalization, dependency, institutionalization, and death [4–7]. Identification of frailty is a major challenge in targeting high risk populations for preventive interventions [2, 8, 9]. Several studies suggest that preventive interventions are capable of decreasing the occurrence of severe loss of independence and/or admission to long-term care geriatric institutions which are critical outcomes for older persons and for society as a whole [8, 10, 11]. Epidemiological studies have shown that several markers can predict the risk of declining independence, institutionalization or death. These markers have been used in developing instruments to identify frailty [6, 9, 12, 13].

Among the instruments available to identify frailty in older persons, the most commonly used are Fried’s Frailty index [6] and Rockwood’s Clinical frailty scale [14]. Both were elaborated from population-based studies data – the Cardiovascular Health Study and Canadian Study for Health and Aging, respectively. The predictive value of these scales has been documented in derivation population samples. Stratification of subjects employing these instruments requires a lengthy process, comprising several questionnaires and complex physical assessments, both requiring high levels of geriatric expertise [9]. Because of these requirements, use of both instruments is limited to specialized teams which means that neither can be transposed to primary care [2, 5, 15]. Another instrument, the Vulnerable Elders Survey-13 (VES-13), also can be employed to predict the risk of functional decline, institutionalization, or death in the elderly, [16, 17]. Developed through analysis of nationally representative sample survey data of elders in the United States [18], the scale is a simple questionnaire comprising 13 items or questions administered during a face-to-face or telephone interview by clinicians or non-clinicians. The scale can also be self-administered. VES-13 questions reflect reliable self-reported health and functional abilities. The survey can be completed in 4–5 min by the elder or his/her proxy. VES-13 provides a score varying from 0 to 10. Subjects scoring 3 or more were found to have four times greater risk of death or functional decline compared to elders scoring 2 or less [17, 18]. The ability of VES-13 to predict functional decline and mortality has been documented in ambulatory patients enrolled in the ACOVE observational survey [17] and older patients in hospital [19], trauma [20], and cancer [21, 22] settings. Because of its simplicity, brevity, and reliability, VES-13 is a promising instrument to screen frailty in various settings including primary care.

The purpose of this study is to translate VES-13 into French and to evaluate the properties of the French version among a sample of elders with a wide variety of functional status.

## Methods

### French translation of VES-13

First VES-13 was translated into French by one of the authors (JB), a French researcher in gerontology whose native language is French. This first French version of VES-13 was then back-translated into English by a second author (JZ), a US researcher in gerontology whose native language is American English. The original VES-13 was then compared to the back-translated version. Discrepancies were examined and resolved to achieve the final French version named “Échelle de Vulnérabilité des Ainés-13” or EVA-13.

### Validation of EVA-13

Feasibility and construct validity were determined by employing EVA-13 with three samples of persons aged 70 or older living in the community with a wide variety of functional status. The first sample was drawn from persons attending a heath prevention center in Paris (Group 1); the second from patients of an ambulatory geriatrics care department outpatient clinic of a hospital (Group 2); the third from inpatients of a hospital rehabilitation care geriatric unit (Group 3). People who attend a health prevention centre are generally in good health, with a low level of disability and good cognitive status. In contrast, patients hospitalized in a hospital’s rehabilitation care units are more likely to have a higher level of disability and impaired cognitive status. The functional status of patients attending an outpatient geriatric clinic is generally better than that of inpatients in geriatric rehabilitation units, but worse than that of people attending a prevention centre. EVA-13 was administrated by one of the authors (LK, a physician training in geriatrics) during face-to-face interviews after a brief explanation of the purpose of the study and agreement by patients to participate. In addition to answering the survey questions, participants were asked if they needed a person to help them carry out activities of daily living (ADLs) and each was administered a Mini Mental Status Examination (MMSE) cognitive assessment. Finally, contact information for participants, his/her proxies, and general practitioner were obtained in order to be able to determine outcomes 18 months later. Subjects with MMSE score below 19 were excluded from the study, and/or if a subject’s answers were obviously incoherent. The percentage of vulnerable elders according to EVA-13 was expected to be lowest in Group 1 and highest in Group 3, with an intermediate value in Group 2.

The ability of VES-13 to predict outcomes was assessed via telephone survey about participants’ living status at 18 months follow-up: alive and living at home, institutionalized, or deceased. First, each subject was called. When the subject could not be reached, his or her proxy was called. If neither participant nor proxy was reached, the general practitioner originally indicated was contacted to determine living status.

Test-retest consistency over time was examined at two different time points. Consistency on the same day was assessed in 15 participants randomly selected among the three groups. Each of them answered all EVA-13 questions posed by one investigator twice on the same day. Consistency on the same week was determined by two different investigators (LK and a nurse not specialized in geriatrics) which administered EVA-13 the same week to the same 15 subjects randomly selected from the three groups. Each investigator independently scored EVA-13 for all 15 subjects; neither was aware of the score assigned by the other investigator. Participants were classified as non-vulnerable (score on EVA-13 of 0–2) or vulnerable (EVA-13 score of 3 or more). Kappa coefficients were calculated to assess classification agreement between the ratings of the test and retest.

### Statistical analysis

The percentage of vulnerable elders was compared between groups using chi-squared tests (Fisher’s exact test). Comparison of continuous variables between groups was carried out by one-way ANOVA, except for EVA-13 scores which were compared by the Kruskal-Wallis test since the variable was not normally distributed. The effect size of the differences of EVA-13 score between the 3 groups was estimated by the Kruskal-Wallis H-test statistics and epsilon-squared estimate [23]. Baseline characteristics of the participants were compared according to the combined outcome variable at follow-up using chi-squared test for categorical variables and t-test for continuous variables, except for EVA-13 scores which were compared using U-Mann and Whitney tests. A logistic regression model was employed to test independence of the variables related to the combined outcome with a *p* value < 0.10 in the univariate analysis. Another multivariate regression analysis was done using the raw score obtained at the EVA-13 against the variables related to the EVA-13 score. Level of significance was determined by a *p* value < 0.05.

## Results

### Translation of VES-13

The process of translation and back translation resulted in two minor mismatches which were resolved by the authors. EVA-13 is presented in the Additional file 1.

### Feasibility and validation study

The distribution of characteristics of the 135 participants is presented in Table 1.

**Table 1:** Characteristics of the participants

Variables	Participants (*N* = 135)	
Age (years, mean +/− SD and range)	81.2 +/− 6.8	70–100
Female gender (n and %)	90	66.7%
Need human help for ADL (n and %)	31	23.1%
Subjects recruited from:		
prevention center (n and %)	45	33.3%
geriatric outpatient clinic (n and %)	40	29.6%
geriatric rehabilitation hospital unit (n and %)	50	37.1%
MMSE score (0–30, mean +/− SD and range)	26.7 +/− 2.8	20–30
EVA-13 total score (mean +/SD and range)	4.13 +/− 3.66	0–10
EVA-13 total score (median and interquartile range)	3	1–8
Non-vulnerable, i.e. EVA-13 score < 3 (n and %)	62	46.0%
Vulnerable, i.e. EVA-13 score > 3 (n and %)	73	54.0%

*ADL* activities of daily living*, MMSE* Mini mental status examination*, EVA-13* Echelle de vulnérabilité des ainés

#### Feasibility

EVA-13 was fully realized with all participants who answered to all items of the scale. We did not record from the participants any questions or misunderstandings about the items of the scale. Time required to complete EVA-13, recorded for the first 20 participants, ranged from 3 to 5 min.

#### Validity

Construct validity, particularly discriminate validity was assessed by exploring whether the scale distinguishes the three groups. As expected, there were significant differences between the three groups for age, MMSE score, and the need for human help with activities of daily living (Table 2). Consistently with our assumptions, the functional status of Group 1 persons was better than that of Group 3 persons and that of Group 2 persons was intermediate (Table 2). We hypothesized that the scale rated the greatest vulnerability level among inpatients of the rehabilitation geriatric unit and the lowest among subjects attending the illness prevention center, with ambulatory geriatric patients expected to be in between these two. The authors found that the percentage of vulnerable elders significantly varied across the three groups consistently to this hypothesis (Table 2). In addition, EVA-13 scores were significantly higher in rehabilitation inpatients with a gradient effect from prevention center subjects to hospitalized patients. The effect size of these differences was estimated by calculating the epsilon-squared estimate at 0.561 corresponding to a Cohen’s d value of 2.259 and very large differences.

**Table 2:** Comparison of characteristics and EVA-13 values between participants recruited from a prevention center (group 1), from a geriatric ambulatory clinic (group 2 and from a geriatric rehabilitation hospital unit (group 3)

Variable	Group 1 (*n* = 45)	Group 2 (*n* = 40)	Group 3 (*n* = 50)	p
Age (years, mean +/− SD)	76.5 +/− 4.8	81.2 +/− 5.5	85.6 +/− 6.6	< 10–4
Female gender (n and %)	29 (64%)	27 (67%)	34 (68%)	0.93
Need human help for ADL (n and %)	1 (2%)	10 (25%)	20 (40%)	< 10–3
MMSE score (0–30, mean +/− SD)	28.6 +/− 1.7	24.7 +/− 2.7	26.6 +/− 2.4	0.009
EVA-13 total score (mean +/SD)	0.91 +/− 1.16	4.27 +/− 3.17	6.90 +/− 3.17	< 10–4*
Non-vulnerable, i.e. EVA-13 score < 3 (n and %)	40 (89%)	17 (42%)	5 (10%)	< 10–3
Vulnerable, i.e. EVA-13 score > 3 (n and %)	5 (11%)	23 (58%)	45 (90%)	< 10–3

*ADL* activities of daily living*, MMSE* Mini mental status examination*, EVA-13* Echelle de vulnérabilité des ainés

*using Kruskal-Wallis test

**Fig. 1:**
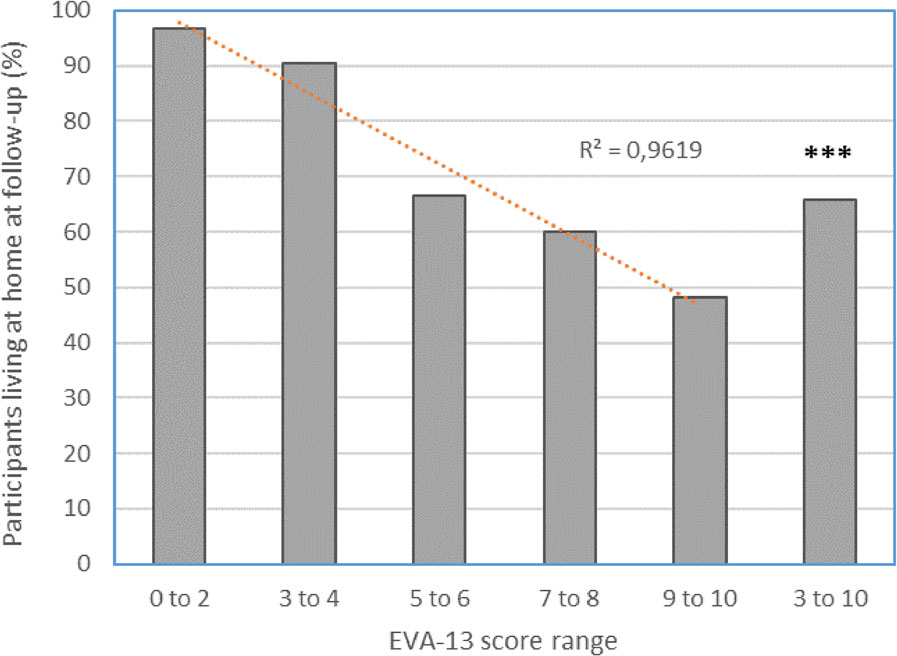
Percentage of subjects being alive at home at follow-up according to the EVA-13 score range. The percentage of subjects considered as vulnerable according the scale (score 3 to 10) were significantly less than that of non-vulnerable subjects (score 0 to 2), with *p* < 10–4

Predictive ability of EVA-13 was assessed by determining living status (living at home, institutionalized, or deceased) of participants at 18 +/− 0.6 months follow-up. At that time, 26 (19%) participants were institutionalized or had died, 104 (77%) were alive and living at home, and 5 (4%) were lost to follow up (3 vulnerable and 2 non-vulnerable). The analysis was conducted with 130 subjects. The percentage of those living at home at follow-up correlated with their EVA-13 score is shown in Fig. 1. Among the 60 non-vulnerable subjects, 58 (96%) were alive and living at home. By contrast, among the 70 subjects with EVA-13 scores of 3 to 10, who were considered vulnerable, 46 (65%) were alive and living at home (*p* < 0.001) (Table 3). EVA-13 scores were significantly correlated to the percentage of subjects alive and living at home at follow-up (*p* < 0.001) (Table 3 and Fig. 1). To examine the value of EVA-13 scores to predict the combined outcome independently from the other variables, the authors performed two logistic regression analyses using living status (living at home / institutionalized or deceased) as the dependent variable. Model 1 included the following variables: age, need for human help with ADLs, group (place of recruitment), and vulnerability status determined by the EVA-13 scale. In Model 1, age, group, and vulnerability status were the three independent variables that related to the dependent outcome variable. For vulnerability the multivariate odd ratio for being institutionalized or deceased at follow-up was 5.55 (95%CI: 1.25–24.71, *p* = 0.024). In Model 2, the raw EVA-13 score was used instead of vulnerable status. In Model 2, age, group, and EVA-13 score were the three independent variables related to the outcome. For the EVA-13 scores, the corresponding multivariate odd ratio was 1.37 (95%CI: 1.14–1.63, *p* = 0.001) per one-point increase in the scale.

**Table 3:** Participants’ characteristics at baseline according to their living status at follow-up

	Outcome at follow-up		p
	Institutionnalized or dead (*n =* 26)	Alive and living at home (*n =* 104)	
Age (years)	87.2 +/− 5.5	79.8 +/− 6.3	< 10–4
Female gender (n, %)	19 (73%)	67 (64%)	0.49
Need human help for ADL (n, %)	11 (42%)	19 (18%)	0.01
Subjects recruited from: (n, %)			< 10–4
prevention center (Group 1)	0	44 (43%)	
geriatric outpatient clinic (Group 2)	7 (27%)	33 (31%)	
rehabilitation hospital unit (Group 3)	19 (73%)	27 (26%)	
MMSE (0–30)	25.9 +/− 2.9	26.9 +/− 2.7	0.12
EVA-13 (0–10)	7.61 +/− 2.81	3.23 +/− 3.30	< 10–4*
Vulnerable, i.e. EVA-13 Score > 3	24 (92%)	46 (44%)	< 10–4

*ADL* activities of daily living*, MMSE* Mini mental status examination*, EVA-13* Echelle de vulnérabilité des ainés

*using U Mann and Whitney test

#### Test-retest

We observed that the agreement between ratings done in the same day in 15 patients (5 from each group). was complete for the classification vulnerable/non-vulnerable (kappa = 1). The two observers independently agreed to consider 6 subjects as non-vulnerable (EVA-13 score < 3) and 8 as vulnerable (EVA-13 score > 3). Disagreement emerged for only one subject with a 1-point difference in the scores assigned to a single question by the two observers. No error in scoring was recorded. The Kappa coefficient was 0.80 indicating good agreement. Test-retest assessment showed a complete agreement between the classification as vulnerable/not vulnerable (kappa = 1).

## Discussion

In this study, the authors developed and examined a French version of the VES-13 instrument – EVA-13 – validating it in a sample of French elders selected from three different settings. Feasibility of EVA-13 was excellent. A gradient in the percentage of vulnerable elders across the groups of participants was observed, with vulnerability frequency the greatest in hospital patients and lowest in subjects attending the health prevention center. Follow-up of participants showed that EVA-13 significantly predicts the risk of mortality and/or institutionalization. These findings obtained in selected older individuals are consistent with those obtained by the original scale in population based samples and in ambulatory older patients included in the ACOVE study and in other cohort studies [17, 19, 24, 25].

In this study design, participants were selected from three different healthcare related settings which resulted in including a larger proportion of vulnerable elders than in population based samples. The authors selected this design to explore construct validity of the translated scale because presently there is no single validated French instrument recognized as a gold standard for identifying frailty in elders. The findings in this study of the French version of the VES-13 are consistent with those obtained with the original scale. However, the odd-ratio values obtained in the predictive validity study should be interpreted with caution because of the selection bias due to the study design.

Our findings confirm that classification into “vulnerable or non-vulnerable” employing the EVA-13 scale is correlated to outcomes of institutionalization and death. Also observed was that the scale’s raw score itself independently correlated with outcomes. These findings are in agreement with the results of Min et al. [19] who observed that an increase of 1 point on the VES-13 scale was associated with a significant increased risk for declining independence. Among elders identified as vulnerable, it is therefore possible to stratify the risk of functional decline.

Our study faces some limitations related to the relatively small number of participants and their very advanced age with the absence of subjects under 70 years of age. In addition, the assessment of test-retest over time was carried out on a limited sub-sample, so its conclusions must be taken with caution.

## Conclusions

The VES-13 scale and its French version – EVA-13 – appear to be promising tools to screen and identify frailty for clinicians including those in primary care. The main advantages of this scale are brevity and availability for use by non-medical health professionals such as caregivers, and telephone interview. The value of this scale for predicting functional decline is comparable to that of frailty instruments based on elder assessments. While VES-13, like any other frailty instrument, cannot directly explain the reason for an elder’s vulnerability, it prompts users of the scale to acknowledge comprehensive geriatric assessment and other investigations to identify the patient’s modifiable vulnerability factors and to select appropriate preventive interventions.

## Supplementary information

**Additional file 1.** Echelle de Vulnérabilité des Ainés-13.

## Data Availability

The dataset supporting the conclusions of the study are available upon request from JB.

## Abbreviations

ACOVE: Assessing care of vulnerable elders
EVA − 13: Echelle de vulnérabilité des ainés-13
MMSE: Mini mental status examination
VES-13: Vulnerable elders survey-13
