# Supplementary material for: Validation of the French version of the Vulnerable Elders Survey-13 (VES-13)
Source: BMC Med Res Methodol. 2020 Feb 5;20:21. doi: 10.1186/s12874-020-0910-x (PMC7003319; doi:10.1186/s12874-020-0910-x)
Supplement: Supplementary file 1 — Additional file 1. Echelle de Vulnérabilité des Ainés-13. [file 12874_2020_910_MOESM1_ESM.docx]

**Additional file 1**

**Echelle de Vulnérabilité des Ainés-13**

**SCORE :** 1 POINT SI AGE DE 75 à 84 ANS

3 POINTS SI AGE > 85 ANS

1. Age ______________________
2. D’une façon générale, par comparaison aux personnes de votre âge, diriez-vous que votre santé est :

- Mauvaise*
- Correcte*

**SCORE :** 1 POINT SI REPONSE MARQUEE * (CORRECTE ou MAUVAISE)

- Bonne
- Très bonne
- Excellente

1. En moyenne, à quel point avez-vous des difficultés pour faire les activités physiques suivantes ?

|  | Aucune difficulté | Légères difficultés | Quelques difficultés | Beaucoup de difficultés | Incapable de le faire |
| --- | --- | --- | --- | --- | --- |
| a. Se pencher, s’accroupir ou se mettre à genoux ?............................................ | 🞎 | 🞎 | 🞎 | 🞎* | 🞎* |
| b. Soulever ou porter des objets d’au moins 4 kg ?......................................... | 🞎 | 🞎 | 🞎 | 🞎* | 🞎* |
| c. Lever ou étendre les bras au-dessus du niveau des épaules ?........................ | 🞎 | 🞎 | 🞎 | 🞎* | 🞎* |
| d. Ecrire, manipuler ou saisir de petits objets ?................................................. | 🞎 | 🞎 | 🞎 | 🞎* | 🞎* |
| e. Marcher 400 mètres ?....................... | 🞎 | 🞎 | 🞎 | 🞎* | 🞎* |
| f. Faire des tâches ménagères difficiles comme frotter les planchers ou laver des vitres ?............................................. | 🞎 | 🞎 | 🞎 | 🞎* | 🞎* |
|  |  |  |  |  |  |

**SCORE :** 1 POINT POUR CHAQUE REPONSE DE LA QUESTION 3 (a à f) MARQUEE *; SCORE MAXIMUM = 2 POINTS

1. Avez-vous quelques difficultés, en raison de votre santé ou de votre condition physique, pour :
   1. Faire vos courses pour des achats personnels (comme des affaires de toilettes ou des médicaments) ?

- OUI 🡪 Avez-vous besoin d’aide pour faire les courses 🞎 OUI* 🞎 NON
- NON
- JE NE LE FAIS PAS 🡪 Est-ce en raison de votre santé ? 🞎 OUI* 🞎 NON
  1. Gérer l’argent (comme suivre ses dépenses ou payer des factures) ?
- OUI 🡪 Avez-vous besoin d’aide pour gérer l’argent 🞎 OUI* 🞎 NON
- NON
- JE NE LE FAIS PAS 🡪 Est-ce en raison de votre santé ? 🞎 OUI* 🞎 NON
  1. Marcher à travers une pièce (l’utilisation d’une canne ou d’un déambulateur est possible) ?
- OUI 🡪 Avez-vous besoin d’aide pour marcher 🞎 OUI* 🞎 NON
- NON
- JE NE LE FAIS PAS 🡪 Est-ce en raison de votre santé ? 🞎 OUI* 🞎 NON
  1. Faire de petites tâches ménagères (comme faire la vaisselle, du rangement, ou du nettoyage) ?
- OUI 🡪 Avez-vous besoin d’aide pour les petites tâches ménagères ? 🞎 OUI* 🞎 NON
- NON
- JE NE LE FAIS PAS 🡪 Est-ce en raison de votre santé ? 🞎 OUI* 🞎 NON
  1. Prendre une douche ou un bain ?
- OUI 🡪 Avez-vous besoin d’aide pour prendre une douche ou un bain ? 🞎 OUI* 🞎 NON
- NON
- JE NE LE FAIS PAS 🡪 Est-ce en raison de votre santé ? 🞎 OUI* 🞎 NON

**SCORE :** 4 POINTS SI UNE OU PLUSIEURS REPONSES DE LA QUESTION 4 (a à e) MARQUEES *
